# Detection of *Campylobacter jejuni* Based on a Real-Time Fluorescence Loop-Mediated Isothermal Amplification Method

**DOI:** 10.1155/2022/3613757

**Published:** 2022-08-31

**Authors:** Ying Liu, Meidi Xu, Jiang Wang, Yang Cao, Tao Wang, Lan Mu, Chao Niu

**Affiliations:** ^1^Tianjin Institute of Environmental and Operational Medicine, Tianjin, China; ^2^College of Basic Medicine, Inner Mongolia Medical University, Hohhot, Inner Mongolia, China

## Abstract

*Campylobacter jejuni* (*C. jejuni*), a foodborne pathogenic bacterium, is among the most prevalent causes of human gastroenteritis globally. We developed and evaluated a loop-mediated isothermal amplification (LAMP) method to detect *C. jejuni*. Outer primers and inner primers were designed based on the *hipO* gene. The ratio between the concentrations of the inner and outer primers and the reaction temperature were then optimized to achieve optimal assay conditions. The analytical specificity tests showed that, among 12 genera of 74 pure bacterial culture strains, only four *C. jejuni* isolates could be detected, whereas no amplification was observed in *C. coli*, *C. lari*, and the other 11 genera of foodborne pathogens (*n* = 70). Moreover, the LAMP assay showed a higher analytical sensitivity (34.2 fg *μ*L^−1^) than the conventional PCR method (342 fg *μ*L^−1^). The limit of detection of *C. jejuni* based on the LAMP assay was 10^3^ CFU g^−1^ in the artificially spiked samples of chicken meat. In conclusion, the developed LAMP assay will be a powerful and practical tool for the fast, specific, and sensitive detection of *C. jejuni*.

## 1. Introduction

The burden of foodborne diseases continues to be a substantial and serious health risk in both developing and developed countries despite the adoption of food safety measures [1]. The high incidence of campylobacteriosis, as well as its duration, makes it highly problematic. The *Campylobacter* genus, which can cause campylobacteriosis, includes the most common pathogens causing gastroenteritis in humans worldwide [2]. The main way in which humans are infected is generally believed to be the consumption of undercooked meat products, as well as raw milk or contaminated dairy products and water [3, 4].

*Campylobacter* spp. infection has been estimated to cause 500 million infections globally every year [5]. Within the genus *Campylobacter*, *C. jejuni* and *C. coli* have been reported to be the most frequent human and animal pathogens [6].

Because of the fastidious and slow growth of *C. jejuni*, conventional culture testing techniques are arduous and time-consuming [7]. In addition, the major concerns are false-negative results and the insufficient sensitivity of the culture-based methods. Presently, nucleic acid-based molecular techniques, such as PCR and qPCR (real-time quantitative PCR), are used to detect foodborne pathogens [8]. However, the need for sophisticated and expensive instruments and the purity of the template DNA prevent them from broad application for routine detection [9]. In addition, there is a cross-reaction between bacterial antigens when serological techniques are used [10].

Loop-mediated isothermal amplification (LAMP) is an innovative technique that amplifies nucleic acid under isothermal conditions taking advantage of autocycling strand displacement behavior of the *Bst* DNA polymerase [11, 12]. LAMP can be run easily in any heating block or dry bath at constant temperature instead of a thermocycler needed in PCR or real-time PCR. Furthermore, LAMP produces approximately 10^3^-fold higher amounts of DNA within an hour than PCR and comparably low susceptibility to inhibitors [9, 13]. Thus, LAMP can be a rapid and simple tool for detecting and identifying foodborne bacterial pathogens.

The selection of target genes determined the analytical specificity of LAMP. The *hipO* gene encoding hippuricase that is exclusively within the *C. jejuni* genome has been used as an identification marker for *C. jejuni* [14]. This study developed a simple, fast, sensitive, and highly specific LAMP assay to detect *C. jejuni* using specific primers designed based on the conserved gene *hipO*. We then evaluated the performance and compared it with conventional PCR. Finally, LAMP was used to distinguish *C. jejuni* from other pathogens in artificially spiked samples of chicken meat.

## 2. Materials and Methods

### 2.1. Bacterial Strains and Culturing

74 isolates, including four *C. jejuni* strains, additional five strains of *Campylobacter* spp., and 65 strains of other 11 species, were used for the analytical specificity testing. Details of the 74 strains are listed in Table 1. *Campylobacter* strains (*C. jejuni*, *C. coli*, and *C. lari*) were grown on Columbia blood agar substituted with 5% sheep blood under microaerophilic environment (85% N_2_, 10% CO_2_, and 5% O_2_) at 42°C for 48 hours. Other strains were cultivated under optimal culture conditions as described previously [15]. *C. jejuni* in the enrichment broth containing the raw chicken samples were isolated and cultured through the method of scribing on the plate.

### 2.2. Genomic DNA Extraction

The genomic DNA used as a template was extracted from a freshly grown bacterial culture following the manufacturer's protocol with a Takara MiniBEST Bacteria Genomic DNA Extraction Kit. 60 raw chicken meat samples including hearts, thighs, skin samples, and wings that were randomly purchased from the traditional market were used. Approximately 2 g chicken meat samples were added to 18 mL of Brucella enrichment broth followed by incubation at 42°C in anaerobic incubators for 30 h. The bacteria were collected by centrifugation at 8000 g for 5 min for subsequent genomic DNA extraction. The quantity and quality of DNA were tested using a NanoDrop spectrophotometer ND-1000 (NanoDrop Technologies, Wilmington, DE). To assess the sensitivity of the LAMP and PCR assays, 10-fold dilution series of gDNA from *C. jejuni* (ATCC33252) starting from 34.2 × 10^0^ ng/*μ*L to 34.2 × 10^−8^ ng/*μ*L was used in the amplification reactions. Furthermore, the DNA from 74 tested isolates used to evaluate the analytical specificity is listed in Table 1.

### 2.3. LAMP Primer Design and LAMP Assay

The online tool PrimerExplorer V4 (http://primer explorer.jp/elamp4.0.0/index.html) was employed. A set of four primers (F3, B3, FIP, and BIP) targeting six sequences on the *hipO* gene according to the sequence database in GenBank (NC_002163.1) was designed. Subsequently, the specificity of primers was determined *in silico* using the BLAST tool (http://www.ncbi.nlm.nih.gov/).  Table 2 lists the sequence, position, and length of the primers. The primer synthesis service was provided by Sangon Biotechnology Co., Ltd. (Shanghai, China). The final optimized LAMP test was performed using the WarmStart LAMP kit containing *Bst* 2.0 WarmStart DNA polymerase (New England Biolabs, USA). A 50x fluorescent dye (New England Biolabs, USA) is also supplied to enable real-time fluorescence measurement of the LAMP amplification. The LAMP reaction was conducted in a 25 *μ*L mixture containing 2.5 *μ*L of 10x LAMP Primer Mix (1.2 *μ*M FIP/BIP, 0.2 *μ*M F3/B3), 12.5 *μ*L of Warm Start LAMP 2x Master Mix, 0.5 *μ*L of 50× fluorescent dye, 2 *μ*L of target gDNA, and 7.5 *μ*L of sterile double distilled water (ddH_2_O) for 60 min at 65°C, followed by 80°C for 5 min, to terminate the reaction on a LightCycler 480 Real-Time PCR System (Roche Applied Science, USA). The melting curve was monitored on the device. The LAMP products were detected visually by turbidity and 2% agarose gel electrophoresis.

### 2.4. PCR Assay

Conventional PCR was carried out with outer primers F3 and B3 (Table 2). The PCR assay was conducted in 25 *μ*L of reaction mixture containing 12.5 *μ*L of 2x Premix master (Takara Taq™ Version 2.0, Takara Biotechnology Co.), 1 *μ*L of 20 *μ*M outer primers F3/B3 each, 1 *μ*L of template genomic DNA (gDNA), and supplementary ddH_2_O. The reaction was carried out using the cycling protocol of 95°C for 5 min and subjected to 30 s at 94°C, 30 s at 55°, and 30 s at 72°C for 35 cycles followed by a final extension cycle for 10 min at 72°C. The amplified products were subjected to 2% agarose gel electrophoresis.

### 2.5. Validation of the LAMP Assay with Spiked Chicken Meat Sample

The assay's detection limit in chicken samples was determined as previously described [15]. Details were as follows. The chicken meat was purchased from a local supermarket. The *C. jejuni* (ATCC33252) strain was freshly prepared on blood agar with cultivation under microaerobic atmosphere conditions as described above. Serial 10-fold dilutions with sterile phosphate-buffered saline were prepared, and colony-forming units were calculated through direct plating. Furthermore, the desired concentration of *C. jejuni* (ATCC33252) pure cultures (10^7^-10^1^ CFU/mL) was spiked onto 25 g of fresh chicken meat. The samples in 225 mL of buffered peptone water (BPW) in a plastic stomacher bag were homogenized with a stomacher. DNA from 1 mL homogenate in chicken samples with bacterial concentrations in a range from 10^7^ to 10^1^ CFU/g was isolated using the DNeasy mericon Food kit (QIAGEN, Germany) as recommended by the manufacturer. These experiments were conducted in triplicate.

## 3. Results

### 3.1. Optimization of Experimental Conditions for the LAMP Assay

Genomic DNA from the *C. jejuni* strain ATCC33252 was used as the template to ascertain optimal reaction conditions of LAMP. First, LAMP assays were carried out with shortage of one or two of the outer and inner primers. There was no amplification in the absence of FIP or BIP primer, while without F3 or B3, the amplification was slightly delayed, and the efficiency decreased when below 65°C for 60 min. Optimal amplification was achieved when both internal primers and external primers were present. Then, the LAMP assays were performed with ratios of inner primers to outer primers ranging from 1 : 2 to 1 : 8. A ratio of 1 : 6 resulted in ideal amplification (Supplementary Figure 1). In addition, reaction mixtures were incubated at different temperatures ranging from 61 to 66°C for 60 min. The optimum temperatures were 65°C and 66°C (Supplementary Figure 2). Therefore, a reaction temperature of 65°C was used for the subsequent experiments.

### 3.2. Analytical Specificity of the LAMP Assay

**Figure 1:**
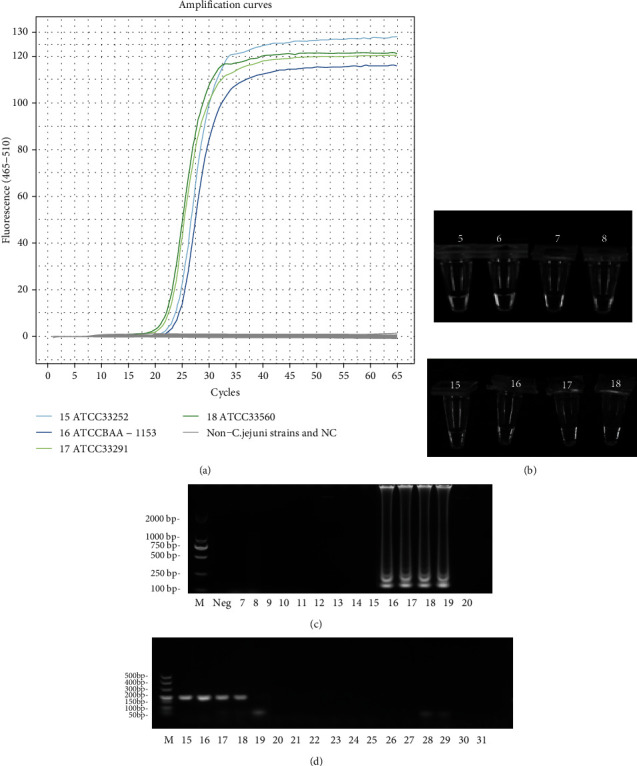
Analytical specificity of the LAMP and PCR for detecting *C. jejuni*. (a) The amplification reaction of 74 bacterial strains was monitored by a real-time PCR System. (b) The result of the LAMP assay was identified with naked eyes. White magnesium pyrophosphate was visualized in the positive amplification (15-18), while no precipitate was found in the negative amplifications (5-8). (c, d) Analytical specificity evaluation of the LAMP assay (c) and conventional PCR (d) by electrophoresis. M: DL2000 marker; M: DL500 marker (Takara). 15: *C. jejuni*, ATCC33252; 16: *C. jejuni*, ATCCBAA-1153; 17: *C. jejuni*, ATCC33291; 18: *C. jejuni*, ATCC33560. Others were the non-*C. jejuni* bacterial strains and negative control (NC) which are listed in Table 1.

74 pure culture reference strains were used to evaluate the analytical specificity (Table 1). All four strains of *C. jejuni* (ATCC33252, ATCCBAA-1153, ATCC33291, and ATCC33560) were detected but not any of the other 70 non-*C. jejuni* strains (Figure 1(a)). Likewise, white precipitate in positive reactions was observed visually, but no visible precipitate was seen in the 70 non-*C. jejuni* LAMP reaction tubes (Figure 1(b)). The amplification products of those positive by LAMP reaction showed typical ladder patterns analyzed by agarose gel electrophoresis (Figure 1(c)). None of the 70 non-*C. jejuni* bacterial strains yielded false-positive results in the assays, indicating their very high analytical specificity. Furthermore, 74 DNA templates were detected by PCR simultaneously, which resulted in a 238 bp *hipO*-specific fragment of *C. jejuni*. No amplicon was obtained from the 70 non-*C. jejuni* strains (Figure 1(d)). The results of PCR were consistent with those of the LAMP assays.

### 3.3. Sensitivity of LAMP Assay

**Figure 2:**
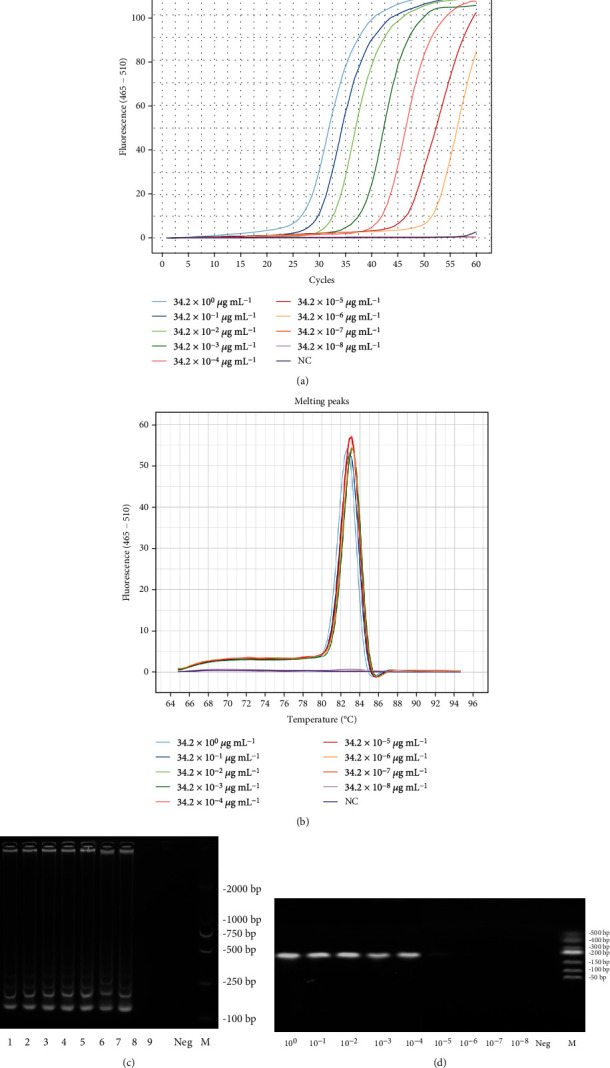
Analytical sensitivity of the LAMP and PCR assay in detecting *C. jejuni*. (a) The sensitivity of the LAMP assay was monitored by a real-time PCR system using serial dilutions of the *C. jejuni* DNA template. NC: negative control. (b) Melting peaks were generated by melting curve analysis. (c) Lanes 1-9: the concentrations used as templates were 34.2 × 10^0^, 34.2 × 10^−1^, 34.2 × 10^−2^, 34.2 × 10^−3^, 34.2 × 10^−4^, 34.2 × 10^−5^, 34.2 × 10^−6^, 34.2 × 10^−7^, and 34.2 × 10^−8^ ng/*μ*L, respectively. M: DL2000 marker. (d) The detection results of the PCR. M: DL500 marker.

The standard strain *C. jejuni* ATCC 33252 was used. The sensitivity of the LAMP and PCR was performed using a 10-fold serial diluted positive DNA template of *C. jejuni* (ATCC33252) with DNA concentrations ranging from 34.2 ng/*μ*L to 34.2 × 10^−8^ ng/*μ*L. The amplification product of LAMP and PCR was detected by real-time fluorescence monitoring and agarose gel electrophoresis, respectively. The results are shown in Figure 2. In the LAMP experiment, the melting temperature (Tm) of seven series (10^0^-10^6^) of the specific amplification was around 82.5°C and therefore was specific (Figure 2(b)). From the products of LAMP, fluorescent amplification curves and multiple DNA bands in agarose gel electrophoresis were observed with ATCC 33252 gDNA from 34.2 × 10^0^ to 34.2 × 10^−6^ ng/*μ*L, but not from 34.2 × 10^−7^ ng/*μ*L (Figures 2(a) and 2(c)). In addition, the 238 bp *hipO* gene amplicons were produced from 34.2 × 10^0^ to 34.2 × 10^−5^ ng/*μ*L, but not from 34.2 × 10^−6^ ng/*μ*L by conventional PCR (Figure 2(d)). In conclusion, the sensitivity of the LAMP assay for *hipO* gene was 34.2 fg *μ*L^−1^ (34.2 × 10^−6^ ng/*μ*L), and that of PCR was 342 fg *μ*L^−1^ (34.2 × 10^−5^ ng/*μ*L) from pure culture, indicating that LAMP showed 10 times greater sensitivity than PCR (Figure 2(d)). We also evaluated the application of established LAMP reaction to detect *C. jejuni* from artificially contaminated samples. In spiked chicken meat samples, the detection limits targeting the *hipO* gene were 10^3^ CFU g^−1^.

### 3.4. Comparison of Using the Real-Time LAMP Assay to Detect *C. jejuni* and Culture-Based Assay Methods in Raw Chicken Meat

A total of 60 chicken meat samples were tested to compare the two methods of detecting *C. jejuni.* 20 *C. jejuni*-positive chicken samples were detected using the culture-based method, whereas 19 samples were detected positive with one as a false-negative case by the LAMP assay (Table 3). Two negative results detected by the LAMP assay were negative in the culture-based assay. Thus, the accuracy, sensitivity, and specificity for the real-time LAMP to detect *C. jejuni* based on *hipO* gene were 95%, 95%, and 95%, respectively. Also, the real-time LAMP had a positive predictive value (PPV) of 90.48% and negative predictive value (NPV) of 97.43%.

## 4. Discussion

*Campylobacter*, a leading cause of foodborne human gastrointestinal diseases, has become a global concern for food safety [6, 16, 17]. The incidence and prevalence of *Campylobacter* infections have increased, causing a substantial burden worldwide compared to the diseases caused by *Escherichia coli* and *Salmonella* [18, 19]. The detection methods have been improving. However, the standard culture method and nucleic acid-based molecular techniques are far from being ideal because they are time-consuming, expensive, labor-intensive, and difficult to perform on-site [12]. Recent studies suggested that LAMP is a practical and efficient tool for the rapid and sensitive detection of *Campylobacter* species [20–22]. Furthermore, the improving fluorescent LAMP assays were evaluated by Yamazaki et al., and a paper-based sensor has been exploited to detect *C. jejuni* and *E. coli* through measuring fluorescence images of the amplicons during LAMP reaction in real time recently, which indicated a robust and high accuracy compared with the turbidimetric LAMP approaches [23, 24].

Several LAMP assays for detecting *C. jejuni* targeting *cj0414* and CJSA_1356 (a unique gene for *C. jejuni* SA clone) have been developed [25–29]. Furthermore, a region of the 16S RNA gene was targeted to detect *Campylobacter* spp. [21, 30, 31]. Besides the *cj0414* gene, the conserved hippurate (*hipO*) gene specific and unique to *C. jejuni* was used as an alternative gene [32]. The analytical specificity of the LAMP targeting the *hipO* gene was evaluated in 74 bacterial strains. The LAMP assay did not detect the *hipO* gene in any 70 non-*C. jejuni* strains, and only the four *C. jejuni* strains were amplified correctly showing high analytical specificity (100%) for *C. jejuni*.

Melting temperature analysis has been used to distinguish different pathogenic bacteria reliably [33]. In this assay, the melting temperatures of *hipO* LAMP products were specific. According to the previous studies, the LAMP assay had an analytical sensitivity of 34.2 fg *μ*L^−1^, and it was 10-fold higher than conventional PCR [34].

As previously reported, the detection limits targeting the *cj0414* gene were 7.9 CFU/tube in the chicken meat, 5.6 × 10^3^ CFU g^−1^ (1.4 CFU per test) in spiked human stool, and 3.89 log CFU g^−1^ and 3.6 CFU g^−1^ in artificially spiked fecal samples [26, 28, 35, 36]. The detection limit of our LAMP assay (34.2 fg *μ*L^−1^) targeting the *hipO* gene was 10^3^ CFU g^−1^ in the spiked samples of chicken meat, whereas 2.5 × 10^2^ CFU mL^−1^ (100 fg *μ*L^−1^) has been reported in the literature [34]. These indicated that the sensitivity of our LAMP assay is comparable to or higher than the sensitivity of previous studies.

## 5. Conclusions

In summary, a sensitive, specific, rapid, and practical LAMP method for *C. jejuni* detection was developed targeting the *hipO* gene even in chicken meat samples. The designed primers based on the *hipO* gene successfully and specifically amplified the target gene from isolated genomic DNA in no more than 90 min, and the analysis was completed using just a water bath, making on-site *C. jejuni* detection feasible. Another important advantage of the LAMP assay is that the result can be judged directly with the naked eye based on white precipitate, which provides results consistent with the gel electrophoresis data. Overall, the LAMP technique for *C. jejuni* detection is a valuable tool in clinical, on-site, and resource-poor settings.

## Figures and Tables

**Table 1:** Genomic DNA of bacterial isolates and results for the LAMP assays.

Bacterial strains	Source	Targeted gene (*hipO*)
Escherichia spp. (*n* = 6)		
*Escherichia coli* O157:H7	ATCC^a^ 35150	−
*Escherichia coli* O157:H7	NCTC^b^ 12900	−
*Escherichia coli* O157:H7	CICC^c^ 21531	−
*Escherichia coli* O26	Stored in our laboratory	−
*Escherichia coli* O138	Stored in our laboratory	−
*Escherichia coli* O139	Stored in our laboratory	−
Salmonella spp. (*n* = 8)		
*Salmonella enterica* subsp. *enterica*	ATCC14028	−
*Salmonella enterica* serovar Choleraesuis	CMCC^d^ 50306	−
*Salmonella enterica* serovar Typhimurium	CMCC50115	−
*Salmonella enterica* serovar Paratyphi	CMCC50774	−
*Salmonella enterica* serovar Rubislaw	CMCC50798	−
*Salmonella enterica* serovar Champaign	CMCC50067	−
*Salmonella enterica* serovar Paratyphi A	CMCC50093	−
*Salmonella enterica* serovar Paratyphi B	CMCC50094	−
Staphylococcus spp. (*n* = 5)		
*Staphylococcus aureus*	ATCC43300	−
*Staphylococcus aureus*	ATCC29213	−
*Staphylococcus aureus*	ATCC27217	−
*Staphylococcus aureus*	ATCC6538	−
*Staphylococcus epidermidis*	ATCC14990	−
Enterococcus spp. (*n* = 3)		
*Enterococcus faecalis*	ATCC19433	−
*Enterococcus faecalis*	ATCC29212	−
*Enterococcus faecalis*	CMCC32001	−
Streptococcus spp. (*n* = 4)		
*Streptococcus pyogenes*	ATCC19615	−
*β-Hemolytic streptococcus*	CMCC32210	−
*Streptococcus pneumoniae*	ATCC49619	−
*Streptococcus thermophilus*	CGMCC1.6472	−
Campylobacter spp. (*n* = 9)		
*Campylobacter jejuni*	ATCC33252	+
*Campylobacter jejuni*	ATCCBAA-1153	+
*Campylobacter jejuni*	ATCC33291	+
*Campylobacter jejuni*	ATCC33560	+
*Campylobacter coli*	ATCC33559	−
*Campylobacter coli*	ATCC BAA-370	−
*Campylobacter coli*	NCTC11366	−
*Campylobacter coli*	CICC23925	−
*Campylobacter lari*	ATCC35223	−
Vibrio spp. (*n* = 9)		
*Vibrio fluvialis*	ATCC33809	−
*Vibrio fluvialis*	CGMCC^e^ 1.1610	−
*Vibrio parahaemolyticus*	ATCC17802	−
*Vibrio parahaemolyticus*	CMCC20502	−
*Vibrio parahaemolyticus*	CMCC20516	−
*Vibrio vulnificus*	ATCC27562	−
*Vibrio vulnificus*	CGMCC1.8674	−
*Vibrio cholerae*	GDMCC^f^ 1.449	−
*Vibrio proteolyticus*	ATCC15338	−
Proteus spp. (*n* = 4)		
*Proteus vulgaris*	CMCC49027	−
*Proteus vulgaris*	ACCC11002	−
*Proteus mirabilis*	CMCC49005	−
*Proteus penneri*	ATCC33519	−
Listeria spp. (*n* = 6)		
*Listeria monocytogenes*	ATCC19118	−
*Listeria monocytogenes*	CMCC54001	−
*Listeria ivanovii*	ATCC19119	−
*Listeria grayi* *Listeria welshimeri*	C12 20031122 GDMCC1.232	−
*Listeria innocua*	ATCC33090	−
Yersinia spp. (*n* = 6)		
*Yersinia enterocolitica*	CMCC52219	−
*Yersinia enterocolitica*	CMCC52206	−
*Yersinia enterocolitica*	ATCC23715	−
*Yersinia enterocolitica*	CMCC52225	−
*Yersinia pseudotuberculosis*	CMCC53504	−
*Yersinia intermedia*	CGMCC1.6197	−
Shigella spp. (*n* = 9)		
*Shigella boydii*	CMCC51515	−
*Shigella boydii*	CMCC51510	−
*Shigella flexneri*	CMCC51508	−
*Shigella flexneri*	ATCC12022	−
*Shigella dysenteriae*	CMCC51135	−
*Shigella dysenteriae*	CMCC51336	−
*Shigella sonnei*	CMCC51424	−
*Shigella sonnei*	CMCC51081	−
*Shigella sonnei*	ATCC25931	−
Other (*n* = 5)		
*Clostridium perfringens*	ATCC13124	−
*Pseudomonas aeruginosa*	ATCC9027	−
*Klebsiella pneumoniae*	CMCC46117	−
*Enterobacter sakazakii*	CMCC45401	−
*Bacillus cereus*	CMCC63302	−

^a^ATCC: American Type Culture Collection, USA; ^b^NCTC: National Collection of Type Cultures, U.K.; ^c^CICC: China Center of Industrial Culture Collection; ^d^CMCC: China Medical Culture Collection; ^e^CGMCC: China General Microbiological Culture Collection Center; ^f^GDMCC: Guangdong Microbial Culture Center.

**Table 2:** The primers for LAMP and PCR in this study.

Assay	Primer	Position	Sequence (5′-3′)	Length
LAMP	F3	21-40	ACTAGACTTACAAGGCGAAT	20
	B3	240-258	TGTGCATTCTTGTAAAGGC	19
	FIP (F1c-F2)	F1c: 98-119 F2: 58-79	TGCGCCACTAATTTTGCAGTAC-CAAATTCATGAAAATCCTGAGC	44
	BIP (B1c-B2)	B1c: 169-189 B2: 219-236	GGCGTTGTGGGGGTTTTAAAA-GCATCCATATCTGCACGA	39
PCR	F (F3)	21-40	ACTAGACTTACAAGGCGAAT	20
	R (B3)	240-258	TGTGCATTCTTGTAAAGGC	19

**Table 3:** Comparison between the LAMP and the traditional culture base method for detection of *C. jejuni* in raw chicken samples (*n* = 60).

Real-time LAMP results	Culture results		Total
	Positive	Negative	
Positive	19	2	21
Negative	1	38	39
Total	20	40	

## Data Availability

The data used to support the findings of this study are available from the corresponding authors upon request.

## Supplementary Materials

Supplementary Materials Supplementary Figure 1: optimization of concentration ratio of inner to outer primers in LAMP experiment. 1-7: the inner and outer primer concentration ratios are 8 : 1, 7 : 1, 6 : 1, 5 : 1, 4 : 1, 3 : 1, and 2 : 1; 8: negative control. Supplementary Figure 2: optimization of the amplification reaction temperature in LAMP. a-f: LAMP amplification curve at 61, 62, 63, 64, 65, and 66°C.
