# Supplementary figures and images for: Detection of Campylobacter jejuni Based on a Real-Time Fluorescence Loop-Mediated Isothermal Amplification Method
Source: Biomed Res Int. 2022 Aug 31;2022:3613757. doi: 10.1155/2022/3613757 (PMC9453007; doi:10.1155/2022/3613757)

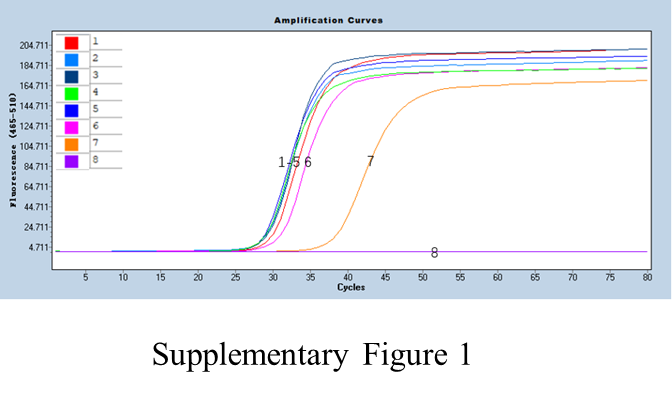

Supplement: Supplementary Materials — Supplementary Figure 1: optimization of concentration ratio of inner to outer primers in LAMP experiment. 1-7: the inner and outer primer concentration ratios are 8 : 1, 7 : 1, 6 : 1, 5 : 1, 4 : 1, 3 : 1, and 2 : 1; 8: negative control. Supplementary Figure 2: optimization of the amplification reaction temperature in LAMP. a-f: LAMP amplification curve at 61, 62, 63, 64, 65, and 66°C. [file 3613757.f1.zip › 3613757.f1.png]

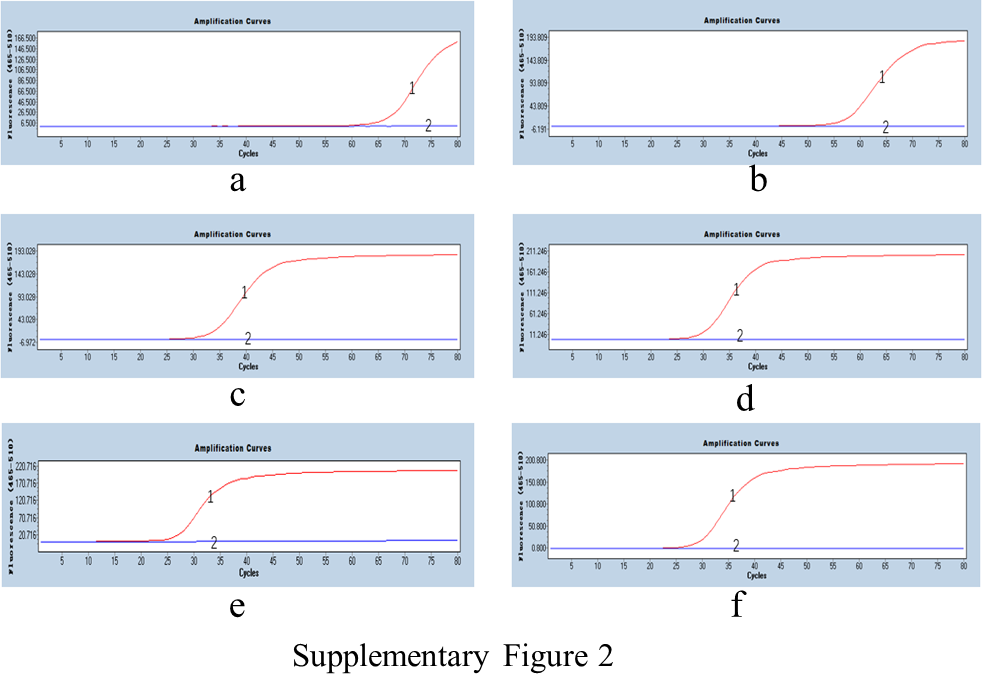

Supplement: Supplementary Materials — Supplementary Figure 1: optimization of concentration ratio of inner to outer primers in LAMP experiment. 1-7: the inner and outer primer concentration ratios are 8 : 1, 7 : 1, 6 : 1, 5 : 1, 4 : 1, 3 : 1, and 2 : 1; 8: negative control. Supplementary Figure 2: optimization of the amplification reaction temperature in LAMP. a-f: LAMP amplification curve at 61, 62, 63, 64, 65, and 66°C. [file 3613757.f1.zip › 3613757.f2.png]
